# What do Portuguese Women Prefer Regarding Vaginal Products? Results from a Cross-Sectional Web-Based Survey

**DOI:** 10.3390/pharmaceutics6040543

**Published:** 2014-10-21

**Authors:** Rita Palmeira-de-Oliveira, Paulo Duarte, Ana Palmeira-de-Oliveira, José das Neves, Maria Helena Amaral, Luiza Breitenfeld, José Martinez-de-Oliveira

**Affiliations:** 1 CICS-UBI: Health Sciences Research Center, Faculty of Health Sciences, University of Beira Interior, Av. Infante D. Henrique, 6200-506 Covilhã, Portugal; E-Mails: apo@fcsaude.ubi.pt (A.P.-d.-O.); luiza@fcsaude.ubi.pt (L.B.); jmo@fcsaude.ubi.pt (J.M.-d.-O.); 2 Pharmacy Department, Centro Hospitalar Cova da Beira, 6200-251 Covilhã, Portugal; 3 Labfit–Health Products Research and Development (HPRD), Lda, Faculty of Health Sciences, University of Beira Interior, Av. Infante D. Henrique, 6200-506 Covilhã, Portugal; 4 NECE–Research Unit in Business Sciences, Faculty of Human and Social Sciences, University of Beira Interior, 6200-209 Covilhã, Portugal; E-Mail: pduarte@ubi.pt; 5 INEB–Instituto de Engenharia Biomédica, University of Porto, 4150-180 Porto, Portugal; E-Mail: j.dasneves@ineb.up.pt; 6 CESPU, Instituto de Investigação e Formação Avançada em Ciências e Tecnologias da Saúde, 4585-116 Gandra PRD, Portugal; 7 Laboratory of Pharmaceutical Technology, Faculty of Pharmacy, University of Porto, 4050-313 Porto, Portugal; E-Mail: hamaral@ff.up.pt; 8 Child and Woman’s Health Department, Centro Hospitalar Cova da Beira, 6200-251 Covilhã, Portugal

**Keywords:** acceptability, vaginal dosage forms, vaginal products, women’s preferences

## Abstract

Therapeutic outcomes of vaginal products depend not only on their ability to deliver drugs to or through the vagina but also on acceptability and correct use. Women’s preferences, in turn, may vary according to age and cultural backgrounds. In this work, an anonymous online survey was completed by 2529 Portuguese women to assess their preferences for physical characteristics and mode of application of vaginal products, according to age. Additionally, intention to use and misconceptions about these issues were assessed. The majority of women of all age groups would use vaginal products to treat or prevent diseases, upon medical prescription. Women preferred vaginal products to be odorless and colorless gels, creams and ointments composed by natural origin drugs/excipients and applied by means of an applicator. Although the majority of women would prefer not to insert any product in the vagina, intention to use for self and recommendation to use for others was associated with previous experiences with vaginal products. General concerns and misconceptions related to use of vaginal products were rare. These data may contribute to the development of products that women are more prone to use.

## 1. Introduction

The vagina has been traditionally used as an advantageous site for drug delivery to achieve either local or systemic effects [1,2]. Antimicrobial, hormonal and spermicidal agents have been widely delivered through this route and many other preventive and therapeutic strategies have been investigated and developed for vaginal delivery [1,3]. Microbicides (*i.e.*, vaginal and rectal topical agents intended to prevent HIV sexual transmission) have gathered particular interest in recent years, although no successful products have yet reached the market. They have been the focus of much research not only on new molecules and their appropriate drug delivery systems, but also on women’s preferences and perspectives concerning these products [4,5]. Interestingly, data on these parameters for the overall group of vaginal products are lacking and the subject seems to have been forgotten. In fact, despite the frequent use of commercial vaginal products, namely to treat vaginal infections which affect up to 75% of women [6,7], data on women’s perceptions concerning currently used products are scarce.

Acceptability of a vaginal product represents a major factor for effectiveness since it clearly influences correct and consistent use, especially when long-term use is required. Most available data on this topic have been obtained from clinical trials [8,9,10], experiences with the use of surrogates (in the case of microbicides) [11,12] and interviews assessing women’s attitudes upon product demonstration (particularly willingness to use or to recommend the product), again, mainly in the context of contraceptives and microbicides [13,14,15] where small samples are frequently evaluated.

Traditional vaginal dosage forms include tablets, capsules and suppositories designed for this application; semisolids such as gels, creams and ointments; foams and liquid preparations. Their use has been associated with insertion difficulties and low residence time resulting in leakage and discomfort [16]. Improvement of vaginal dosage forms has been achieved by either developing formulations with increased residence time (mainly by using bioadhesive polymers) or new delivery systems such as rings and films (thin polymeric strips that rapidly dissolve/disperse in contact with vaginal fluids) [3,16,17].

The development of more appropriate vaginal products must consider women’s preferences that may, in turn, depend on their age, socio-economic status and cultural backgrounds [5,18] and on the type of product they would need to use. For products used for contraception or to treat vaginal infections, opinions collected from reproductive aged women would be preferred, while for hormonal replacement therapy gathering the opinion of older women would be essential. Also, women’s fears and misconceptions on the safety of these products should be addressed by prescribers and educational strategies should be adopted to assure compliance and therapeutic success.

We conducted a large scale online survey to assess Portuguese women’s experiences, preferences and general perspectives on the vaginal route for drug delivery and on vaginal products [19]. In this work, women’s preferences on the physical properties and mode of insertion of vaginal products are analyzed according to age groups. Additionally, misconceptions and fears related with the use of vaginal products are assessed and results are compared with data available from previous studies conducted across other populations [13,18,20,21].

## 2. Experimental Section

### 2.1. Type of Study and Questionnaire Development

A descriptive cross-sectional study was conducted using a web-based survey. A questionnaire was developed with two main goals. The first was to collect data on previous use of genital products, personal preferences for dosage forms and general perceptions on the advantages/disadvantages of the vagina as a route for drug delivery [19]. The second one was to assess women’s preferences regarding the general characteristics of vaginal products along with their fears, misconceptions and personal perspectives on vaginal products. The scope of this work is to analyze the second goal of the survey.

The questionnaire comprised questions designed for multiple-choice answers, binary answers (Yes/No) and five-point Likert scales anchored at 1 = {I definitely wouldn’t use the product}/{I strongly disagree} and 5 = {I would definitely use the product}/{I strongly agree} to evaluate women preference and intention to use. Answers to some questions were pointed as essential for the questionnaire to be considered valid. Blank answers were possible for the remaining ones. Furthermore, 12 questions were included for demographic and general gynecological/obstetric previous history characterization (questions applied within the scope of this work have been translated from Portuguese and are available in the Supplementary Information, Part I).

To assess women’s fears and misconceptions regarding vaginal products and the vaginal route, women were asked to state their level of {agreement} or {disagreement} with sentences developed to specifically address fears of not being able to remove products after insertion in the vagina and preconceived ideas of vaginal products interference with virginity. Also, statements were included to assess women’s general perspectives on the safety of vaginal products and willingness to reuse or recommend vaginal products to other women (Supplementary Information, Part I).

An introductory text was included in the online survey to assure that participants received sufficient information on the objectives of the study and on confidentiality and anonymity of research gathered data. At the end of the questionnaire, researchers’ contacts were made available for any further information, suggestions or requests of published results.

The questionnaire was pre-tested in 10 women in order to detect structure errors and interpretation difficulties. Following this initial step, corrections and suggestions were adopted. The final questionnaire and the methodology of the study were approved by the Ethics Committee of the Faculty of Health Sciences (CE-FCS-2012-031, 6 February 2013), University of Beira Interior (Covilhã, Portugal).

### 2.2. Study Population, Recruitment and Sample

The target population was defined as being represented by Portuguese women aged 18–65 years old. Anonymous answers were collected from February to May 2013. Women were contacted by email, which included the questionnaire’s web link and using different organizational email lists (societies, women’s groups, universities, and others). The questionnaire was also publicized in Internet social networks. No incentives were used. The geographic distribution of answers was monitored throughout the study and compared with the Portuguese demographic distribution data from the last (2011) Official National Census [22,23].

The final sample of the study was composed by 2529 complete questionnaires received. Geographical distribution of respondents was not overly represented when compared with demographic data, with the exception of Castelo Branco district as result of being the home district of our University (detailed information on geographic distribution across Portuguese continental districts and autonomous regions is shown in Supplementary Information Part II, Table S1).

In order to allow for differential data analysis concerning age, the following groups were defined: 18–24 (comprising 427 women), 25–34 (885 women), 35–44 (714 women), 45–54 (342 women), and 55–65 (161 women). Detailed sample characterization data are shown in Supplementary Information (Part III, Table S2).

### 2.3. Statistical Analysis

Descriptive statistical analysis was performed and the *t*-test was used to determine if significant differences existed among age groups’ responses to various survey questions. Additionally two tailed *t*-test was used to compare the mean score of each group with the middle point of the scale in order to assess if the group’s opinion was significantly different from the indifference point. One-way ANOVA and Tukey *post-hoc* multiple comparison tests were used to compare women’s preferences on products’ characteristics (defined color, intensity of odor, presence or absence of flavor, *etc.*) for each age group. Additionally, the chi-square test was used to assess differences in preferences for vaginal dosage forms and on the general properties considered relevant for vaginal products, among age groups. A confidence level of 95% was established for all inferential analyzes. All tests were performed using SPSS Software (version 21, SPSS Inc., Chicago, IL, USA).

## 3. Results and Discussion

Commercially available products for vaginal use vary widely in nature and purpose ranging from simple collection of menstrual fluid to treatment of symptoms and diseases [24,25]. Although prescribed vaginal therapeutics have been traditionally dependent on physician’s preferences, women’s participation in the treatment decision, particularly concerning the route of administration, is currently being actively promoted [25]. On the other hand, for over-the-counter (OTC) products, the choice relies almost completely on women’s preferences.

Through this web-survey women’s preferences on the physical properties and mode of insertion of vaginal products were assessed along with fears and misconceptions regarding these products and the vaginal route.

Concerning the proportion of women that would use vaginal products for each of their most common purposes (Table 1), the majority (82%–87%), in all age groups, selected the option that considered the vagina for drug delivery following medical prescription. For women in the reproductive age (<45), this proportion was even similar to the one obtained for products for menstrual use (Table 1). A previous international population study, comprising 9441 women (18–44 years old) and intended to assess their attitudes, perceptions and knowledge about the vagina, reported that up to 75% of women had experienced a vaginal problem, although only 35% (27% among 691 Portuguese women participating in the study) perceived the vagina as a site for drug delivery [21]. Results from our study indicate that women’s knowledge on this topic may have evolved from 2004 to date. Also, improved access to information about vaginal products may be associated with the high educational level that characterizes our sample (Supplementary Information Part I, Table S1) although comparisons with the previous study cannot be performed since the educational level of the sample was not reported.

Interestingly only 20%–25% of women in each age group reported that they would use the vaginal route to treat or prevent diseases even without medical prescription. OTC products for the treatment of vaginal conditions are widely available in Europe and in the USA [24,26,27] and their frequent use has been reported in several studies, particularly for yeast infections [26,27,28,29] and for the relief of vaginal atrophy associated symptoms [30,31]. It is, therefore, possible that a higher response rate could be obtained in this survey if the objective of the question had been to gather knowledge on specific health conditions instead of on generic concepts and diseases, which are usually related to medical consultation.

Approximately one third of women aged less than 45 stated they would be prone to use a vaginal contraceptive method (Table 1). Lower response rates obtained from older women are probably associated with the fact that they may not have selected options they would not need to use in view of their physiological status, as is the case of menstrual hygiene products.

Interesting data were obtained for sexual lubrication that was highly referred by women of all age groups with no differences between younger (18–24) and older (55–65) individuals. In fact, although vaginal dryness has been reported in adult women of all ages [32] it is more likely to occur as a consequence of vaginal atrophy caused by the decreased production of sexual hormones after menopause, causing dyspareunia that usually requires the use of lubricants [31]. Nevertheless, lubricants are also used to facilitate or enhance the sexual experience independently of dyspareunia or other atrophy related symptoms [33,34]. Despite this, sexual stimulation was scarcely selected as a possible vaginal product application.

**Table 1:** Selected conditions for use of vaginal products and their relevant characteristics, according to women’s age (total *n* = 2529).

Queries	Age Groups (% within Group)					
	18–24	25–34	35–44	45–54	55–65	Total Sample
***In which of the following situations would you apply a product in the vagina?***						
Contraception	34.7	34.6	30.0	22.9	25.3	30.1
Treatment/prevention of diseases following medical prescription	84.6	86.3	87.4	82.5	82.7	82.8
Treatment/prevention of diseases even without medical prescription	25.8	27.0	19.0	18.7	15.3	21.9
Menstrual hygienic purposes (tampon, cup)	87.0	82.1	74.5	65.4	58.7	74.5
Sexual lubrication	49.9	52.4	47.1	42.8	53.3	47.6
Sexual stimulation	24.1	22.4	20.4	18.1	18.0	20.6
***Which characteristics of a vaginal product do you consider relevant?***						
Color	15.7	15.5	11.9	12.6	15.5	14.1
Odor *	64.4	62.0	58.5	51.5	44.7	58.9
Flavor *	9.8	5.8	6.4	3.5	1.2	6.1
Origin of ingredients (natural/synthetic)	45.2	38.4	41.2	40.9	39.8	40.8
***What dosage form would you prefer for an intravaginal medication?*** (more than one answer could be selected)						
Vaginal tablet/capsule *	48.0	42.3	37.6	46.8	43.5	42.5
Vaginal suppository *	33.2	48.7	50.7	55.0	53.4	47.7
Gel/cream/ointment *	74.8	65.0	62.1	59.4	60.2	64.6
Irrigation solution *	36.0	26.7	23.7	29.5	19.9	27.3
Foam	26.1	21.4	19.9	20.5	17.4	21.3
Ring *	11.8	14.8	10.4	9.1	3.7	11.5
Film	5.6	5.4	3.9	4.1	3.1	4.7

* denotes statistical significant differences (*p* < 0.05; chi-square test) among age groups.

The physical characteristics of a vaginal product, including its sensorial properties and its mode of application are strong determinants of women’s acceptability [13,20] thus influencing consistent use and consequent therapeutic efficacy. Semisolid dosage forms (gels, creams and ointments) were selected as the most preferred dosage forms among all age groups with a higher preference among younger women (*p* < 0.05). Detailed data on differential acceptability among semisolid dosage forms were not collected since their classification is not easily perceived by the majority of users. Gels and creams have been referred as the most acceptable dosage forms either following experiences with the product (actual vaginal use or simple handling upon demonstration) [12,20] or as an idealized vaginal product [18], particularly due to easiness and comfort during insertion. However leakage and messiness have been frequently pointed out as disadvantages [12,35]. Comfort issues may also explain the lower acceptability of vaginal suppositories among younger women when compared with the other age groups (Table 1) since these dosage forms are usually bigger than tablets and may be perceived as more difficult to insert. Hardy *et al.* reported similar results for comparative preferences for suppositories among adolescent and adult Brazilian women in the context of contraceptive products [18,20].

It is not surprising that less known vaginal dosage forms or devices gather less acceptability. The vaginal ring, for example, is currently available in Portugal only for contraception purposes. The fact that only 10%–15% of women under 45 years old selected the vaginal ring may be associated with a limited knowledge on the characteristics and potential advantages of these devices since they are relatively recent. Nevertheless, considering that around 30% of women within these age groups stated that they would choose the vaginal route for contraception, our data indicate that almost one third of these women (30%) would be prone to use the vaginal ring. In contrast, the very low preference rate obtained among older women indicates that these women could be less prone to use a product specifically designed for their needs, such as a hormonal ring for vaginal atrophy probably as a consequence of having had no experience in using them and having received scarce information on vaginal rings. In clinical trials, this dosage form has been considered comfortable and easy to use [9] and it has even been preferred by women when compared to creams, suppositories and tablets for symptomatic relief in vaginal atrophy [36]. Its prolonged therapeutic effect (weeks to months) upon placement into the vagina is also highly appreciated by women, which can also abbreviate compliance issues. The vaginal film is a dosage form that is not currently available on the Portuguese market, which makes it highly unlikely that the women surveyed would have previous experiences with it. For the purpose of this survey, a photograph of the dosage form was shown to support the question. So, only a small percentage of women revealed intention to use a film. Although films have been associated with several advantages over traditional dosage forms, such as rapid dissolution/dispersion following contact with vaginal secretions and avoidance of leakage and messiness [12,17], our data showed that, as in the case of vaginal rings for older women, their potentials and handling must be appropriately communicated to women in case a film product will be developed for the Portuguese market.

When women were asked about the sensorial properties that they would consider relevant for these products, odor was the most selected by all age groups with a predominance of relevancy for younger women (18–24) when compared with the oldest women (55–65). The origin of drugs/excipients was considered much more relevant than color while flavor was only scarcely chosen (Table 1). Product flavor is usually regarded important in relation to sexual intercourse, namely female oral sex [37]. Although no data was collected regarding this issue, poor relevance of flavor may indicate that most women do not use or avoid using vaginal products when such practices take place.

**Figure 1:**
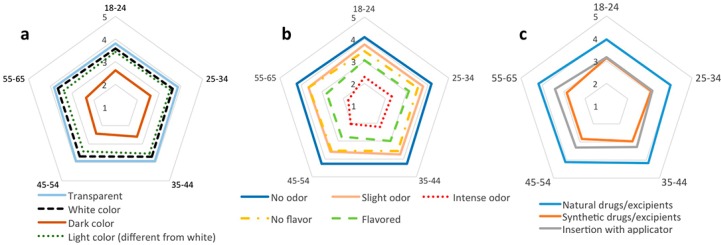
Women’s preferences on (**a**) color, (**b**) odor and flavor, (**c**) origin of ingredients and mode of insertion of idealized vaginal products according to age groups. Data are expressed as the mean value for each age group based on the five-point Likert scale ranging from 1-{I would definitely NOT use the product} to 5-{I would definitely use the product}. The number of respondents for this specific question, in each age group, was: 422 (18–24); 853 (25–34); 675 (35–44); 290 (45–54); 118 (55–65).

Further characterization on these preferences for vaginal products properties was achieved by using Likert scales (Figure 1). Preferences for each specific characteristic were shown to be generally different from neutrality in all age groups, except for the presence of flavor in age groups 18–24 and 35–44 and for the synthetic origin of ingredients in age group 35–44 and 55–65 (*p* > 0.05) which means that, for these women, the referred characteristics are considered indifferent.

Women from all age groups clearly preferred vaginal products to be transparent, or eventually white or light colored, while dark colored products were rejected by the majority of women (Figure 1a). Preferences of women aged over 45 were statistically different from those aged 25–34 in their more negative reaction to a dark colored product as shown by Tukey *post-hoc* multiple group comparison (*p* < 0.05). However, the fact that the age groups are not equally sized may limit further conclusions on age differences. As previously stated, odor was identified as the most important sensorial characteristic under these women’s perspectives (Figure 1b). The majority of them would definitely use a product with no odor. Intense odor was not well accepted by any of these age groups with both groups of older women (>45) differing from both groups of younger women in their lower acceptability for a product with this characteristic (*p* < 0.05). Flavor was the least important sensorial characteristic. For women aged 18–24 and 35–44 the presence of flavor in a vaginal product was indifferent (no difference between the obtained mean value and the score 3 as tested by *t*-test; *p* > 0.05) while for the majority of other women it was even considered negative. Concerning the mode of insertion, women in all age groups revealed an average preference for using an applicator (Figure 1c). Hardy* et al.* generally reported similar preferences among Brazilian women (*n* = 635) aged 18–45 in the context of vaginal contraceptives, ending by defining a preferred product by having no color (or with light colors), no odor and no taste, and that would be applied by means of an applicator [18]. Although differences in acceptability according to cultural backgrounds have been reported in comparative studies [14,38] these results indicate that some general perspectives on vaginal products may be shared by women, at least, in developed countries. In fact, data on the acceptability of the combined contraceptive vaginal ring obtained from multi-center clinical trials (conducted in Canada, USA and 12 European countries) further support this perspective [9].

Since plant-derived treatments are increasingly being proposed as alternative treatments for vaginal ailments [39,40], women were also asked on their preferences for the origin of drugs and excipients. Interestingly, it was evident that the majority of women in all groups preferred products based on natural drugs/excipients. The use of synthetic origin drugs and excipients was considered either indifferent, slightly negative (by women aged more than 45) or even slightly positive (18–24). The perception by the general population that natural products are safer may account for these observations [41]. Therefore, more important than complying with the preference for ingredients of natural origin, formulators should be particularly careful with safety aspects of vaginal products [42].

**Figure 2:**
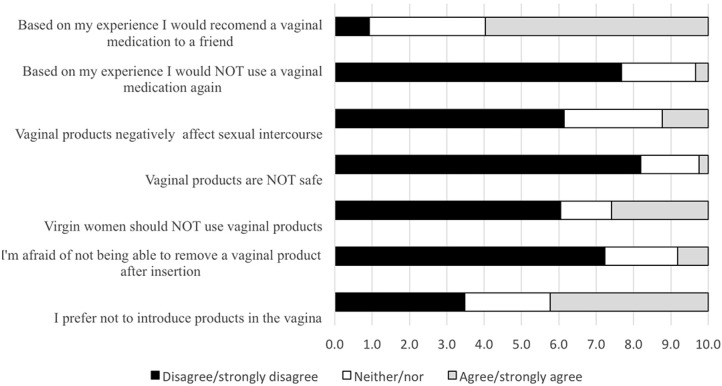
Women’s fears, misconceptions and personal perspectives on vaginal products. Bars represent the proportion of women that disagree, neither agree nor disagree, or agree with each sentence (*n* = 2529).

Assessment of women’s taboos and concerns related to vaginal products revealed that the majority of women disagreed with statements associated with misconceptions on the safety of vaginal products (Figure 2). Since the majority of these women (85%) had previously used at least one intravaginal product, mainly to treat vulvovaginal infections (75%) these results may be associated with positive experiences with those products [19]. Also these may indicate that women had previously received adequate information on vaginal products and on the vaginal route.

Although 42% of women prefer not to use vaginal products, this preference does not seem to hinder vaginal products use, if needed, based on women’s own previous experience. This has been further confirmed by the fact that the majority of women (60%) would even recommend the use of a therapeutic or preventing vaginal product based on their previous experience. Again, when compared with data previously published by Nappi *et al.* [21] these data point to positive previous experiences or to higher levels of information and knowledge that may be associated with the educational level of the sample or with the expected evolution on consumer information. For example, in that study, 71% of all women (74% of Portuguese participating women) believed that {a tampon could get lost or trapped inside the vagina}, (although up to 73% used tampons either regularly or sometimes) [21]. In our study only 10% were {afraid of not being able to remove a vaginal product after insertion}. Also, perception of the safety of vaginal products is particularly high thus indicating women’s confidence on these type of products.

The major strength of this work is the high number of women surveyed and the fact that it focuses on acceptability of vaginal products in a real life context. In fact, women were questioned about their preferences regarding products that they may have used or may need to use in view of the high prevalence of vaginal health problems, instead of an exercise on a hypothetical product for indications not yet available.

This study also presents some limitations. The fact that only Portuguese women were surveyed limits the translation of the conclusions to other populations. However, as previously discussed, some of our results are similar to those available from other countries, suggesting that the generalization of results could be considered at least for women from other developed nations.

The option for the web-based survey instead of a paper-based or interview-based methodology may also be regarded as a limitation since those erroneous answers that may have occurred are difficult to identify due to the absence of direct contact between researchers and participants. In addition, the sample excludes women who are not Internet users. Although these may represent disadvantages of this methodology, results from previous studies comparing web-based and paper-based surveys suggest that the two methods are comparable regarding data reliability and validity [43,44,45]. Moreover, the web-based survey methodology allows for reaching a large sample of respondents while maintaining anonymity which can be important when sensitive topics are the focus of the survey [46], as is the case of sexuality related issues [21]. It is also cost-saving.

## 4. Conclusions

Women’s acceptability for currently available vaginal products has been frequently overlooked although it represents an important factor for compliance and therapeutic success. This study, the largest on this specific subject, allowed for the identification of Portuguese women’s positive perspectives on the vaginal route of drug delivery and on the use of vaginal products with rare misconceptions regarding these issues. The intention to use the vagina as a route for drug delivery was clearly pointed out by all age groups, indicating high levels of information and denoting very few fears or misconceptions related with the products applied on or through this route of administration. Characteristics of idealized vaginal products were generally shared by women of all ages consisting of odorless and colorless semisolids, composed by natural drugs/excipients and applied by means of an applicator.

These data may contribute to the design of vaginal products that best fulfill women’s preferences to gather increased compliance and consequent efficacy. Further studies on women’s acceptability for specific products shall consider the therapeutic application and the age group targeted for their use.

## Supplementary Files

Supplementary File 1
