# Supplementary material for: What do Portuguese Women Prefer Regarding Vaginal Products? Results from a Cross-Sectional Web-Based Survey
Source: Pharmaceutics. 2014 Oct 21;6(4):543–56. doi: 10.3390/pharmaceutics6040543 (PMC4279132; doi:10.3390/pharmaceutics6040543)
Supplement: Supplementary File 1 [file pharmaceutics-06-00543-s001.pdf]

Supplementary Information

PART I

List of questions applied (translated from Portuguese) within the scope of this work. Instructions and figures are not shown. Questions marked with "*" were mandatory.

1—If you were prescribed an intravaginal medication what dosage form would you prefer? (please select more than one if you wish, including those that you have never tried but would accept to try) *

1.1—Vaginal tablet/capsule
1.2—Vaginal suppositories
1.3—Gel/cream/ointment
1.4—Vaginal irrigation solution
1.5—Vaginal foam
1.6—Vaginal ring
1.7—Vaginal film
1.8—Other. Please specify_______

2—In which of the following situations would you apply a product in the vagina? (if you wouldn't apply a product in the vagina in any situation, please do not answer this question)

2.1—Contraception (vaginal ring, diaphragm, spermicides, *etc*)
2.2—Treatment or prevention of infections following medical prescription
2.3—Treatment or prevention of infections even without medical prescription
2.4—Hygienic purposes associated with menstruation (tampon, menstrual cup)
2.5—Sexual lubrication
2.6—Sexual stimulation
2.7—Other. Please specify_______

3—Which characteristics of a vaginal product do you consider relevant? (do not answer the question if consider none of the following characteristics to be relevant for vaginal products)

3.1—Color
3.2—Odor
3.3—Flavor
3.4—Origin of excipients/drugs (natural or synthetic).

4—Considering the descriptions presented below, what would be the probability for you to use a vaginal product presenting each of the specified characteristics, if you could choose them:

Statements	I definitely wouldn't use it (1)	I probably wouldn't use it (2)	Neutral (3)	I would probably use it (4)	I would definitely use it (5)
No color (transparent)	( )	( )	( )	( )	( )
White color	( )	( )	( )	( )	( )
Dark color	( )	( )	( )	( )	( )
Light color (different from white)	( )	( )	( )	( )	( )
No odor	( )	( )	( )	( )	( )
Slight odor	( )	( )	( )	( )	( )
Intense odor	( )	( )	( )	( )	( )
No flavor	( )	( )	( )	( )	( )
Flavored	( )	( )	( )	( )	( )
Natural origin ingredients	( )	( )	( )	( )	( )
Synthetic ingredients	( )	( )	( )	( )	( )

5—Please consider the following statements and select the option that best describes your opinion *:

Statements	Strongly disagree (1)	Disagree (2)	Neither agree nor disagree (3)	Agree (4)	Strongly agree (5)
I prefer not to introduce products in the vagina	( )	( )	( )	( )	( )
Women shouldn't use vaginal products before the first sexual intercourse	( )	( )	( )	( )	( )
I am afraid of not being able to remove a device (tampon, vaginal ring, menstrual cup, *etc.*) from my vagina	( )	( )	( )	( )	( )
Vaginal products are NOT safe	( )	( )	( )	( )	( )
Vaginal products negatively affect sexual intercourses	( )	( )	( )	( )	( )
If I must apply a product in my vagina I prefer to use an applicator instead of my fingers	( )	( )	( )	( )	( )
Based on my experience I wouldn't use a vaginal medication (intended to treat or prevent a disease)	( )	( )	( )	( )	( )
Based on my experience I would recommend the use of a vaginal medication (intended to treat or prevent a disease) to a friend	( )	( )	( )	( )	( )

RESPONDENT CHARACTERIZATION

1—In what year were you born?
2—Where do you live? (If you are currently living outside Portugal please select the option that best identifies the place where you lived in Portugal).

2.1—In a city (*women who selected this option were considered urban*)
2.2—In a town (*women who selected this option were considered rural)*
2.3—In a village (*women who selected this option were considered rural)*

3—Identify the district where you live
4—Please select your educational level

4.1—Less than high school
4.2—High school
4.3—Technical school
4.4—University degree (*field of knowledge*)
4.5—Master degree
4.6—PhD or higher

5—Do you have regular sexual intercourses?
6—Do you use any contraceptive method?
7—If your answer is YES, which contraceptive method do you use?

7.1—Pill
7.2—Intrauterine device
7.3—Implant
7.4—Male condom
7.5—Female condom
7.6—Spermicide
7.7—Diaphragm
7.8—Ring
7.9—Other. Please specify_______

8—Do you visit a gynecologist?
9—If your answer is YES, how often do you visit the gynecologist?

9.1—Only when you are ill
9.2—1-2/year
9.3—Routine consultation
9.4—Other. Please specify_______

10—Have you ever been pregnant?
11—If your answer is YES, how many vaginal deliveries or cesareans have you undergone?
12—Are you menopausal or pre-menopausal?

PART II

Table S1. Geographical distribution of respondents (n = 2529) across Portuguese continental districts and autonomous regions and comparison with female population density according to the last National Census (2011) (data available at www.pordata.pt and www.ine.pt).

District/Autonomous Region (AR)	% Female Resident Population	% Respondents
Aveiro	6.7	5.9
Beja	1.4	0.9
Braga	8.0	4.9
Bragança	1.3	0.8
Castelo Branco	1.9	12.6
Coimbra	4.1	4.7
Faro	4.2	2.8
Guarda	1.5	2.0
Évora	1.6	0.9
Lisbon	21.5	27.0
Leiria	4.4	3.5
Portalegre	1.1	0.6
Oporto	17.2	17.4
Santarém	4.3	3.5
Setúbal	8.0	5.5
Viana do Castelo	2.4	1.2
Vila Real	2.0	1.0
Viseu	3.6	2.0
Azores (A.R.)	2.3	1.9
Madeira (A.R.)	2.5	1.0

PART III

Table S2. Sample characterization: demographic data and gynecological/obstetric general history.

Variable	Categories	*n* (2529)	%
Age	18–24	427/2529	16.9
	25–34	885/2529	35.0
	35–44	714/2529	28.2
	45–54	342/2529	13.5
	55–65	161/2529	6.4
Mean ± SD	35.6 ± 10.7		
Educational Level	Less than high school	21/2523	0.6
	High school	322/2523	12.8
	Technical school	104/2523	4.1
	University degree	1164/2523	46.1
	Master degree	693/2523	27.5
	PhD or higher	219/2523	8.7

Table S2. *Cont.*

Variable	Categories	*n* (2529)	%
**Address region**	Urban	1997/2522	79.2
	Rural *	525/2522	20.8
**Birth delivery**	Vaginal	728/2529	28.8
	Cesarean	368/2529	14.6
**Regular sexual activity**	Yes	2087/2502	83.4
	No	415/2502	16.6
**Women using contraception**		1807/2507	72.1
**Current contraceptive methods**	Oral contraceptives	1231/1809	68.0
	Patch	19/1809	1.1
	Intrauterine device	153/1809	8.5
	Implant	28/1809	1.5
	Male condom	700/1809	38.7
	Female condom	15/1809	0.8
	Spermicide	10/1809	0.6
	Diaphragm	6/1809	0.3
	Ring	39/1809	2.2
	Tubal occlusion	4/1809	0.2
	Coitus interruptus	1/1809	0.1
**Gynecological follow-up frequency**	When ill	168/2162	7.8
	1–2/year	962/2162	44.5
	Routine consultation	1009/2162	46.7
	Other (more frequently or rarely)	23/2162	1.1
**Menopause**	Yes	327/2504	12.9

* women living in small towns or villages were considered rural.

© 2014 by the authors; licensee MDPI, Basel, Switzerland. This article is an open access article distributed under the terms and conditions of the Creative Commons Attribution license (http://creativecommons.org/licenses/by/4.0/).
